# A LASSO-based nomogram for predicting acute bilirubin encephalopathy in newborns with severe hyperbilirubinemia

**DOI:** 10.3389/fped.2026.1818387

**Published:** 2026-05-15

**Authors:** Qifa Hu, Zhenzhu Yao, Haixia Zhu, Xue Feng, Hongxing Li

**Affiliations:** 1 Department of Pediatrics, Huidong County People's Hospital, Huizhou, Guangdong, China; 2 Department of Neonatology, Shenzhen Children's Hospital, Shenzhen, Guangdong, China; 3 Department of Gastroenterology, Children's Hospital Affiliated to Zhengzhou University, Henan Children's Hospital, Zhengzhou, Henan, China

**Keywords:** acute bilirubin encephalopathy, hyperbilirubinemia, LASSO, newborns, nomogram

## Abstract

**Background:**

To develop and validate a nomogram for predicting acute bilirubin encephalopathy (ABE) in newborns with severe hyperbilirubinemia.

**Methods:**

A retrospective analysis was conducted on 287 newborns with severe hyperbilirubinemia who visited the neonatal department of Shenzhen Children's Hospital from January 2015 to December 2022. A simple random sampling method was used to divide the subjects into a training group (200 cases) and a validation group (87 cases) at a ratio of 7:3, collecting general information and biochemical indicators of the neonates. LASSO regression and cross-validation were performed using RStudio (4.2.3) to select optimal predictors. A multivariate logistic regression model was then constructed and visualized as a nomogram. Model performance was evaluated using receiver operating characteristic (ROC) curves, calibration plots, and decision curve analysis (DCA).

**Results:**

LASSO regression combined with multivariate logistic analysis identified six potential predictors selected by LASSO. Among these, four were independently associated with ABE in the multivariate model: delivery method (OR = 3.563, 95%CI: 1.391–9.145), birth trauma-related hemorrhage (OR = 3.024, 95%CI: 1.156–7.816), total bilirubin (OR = 1.012, 95%CI: 1.006–1.019), and reticulocyte percentage (OR = 1.185, 95%CI: 1.019–1.478) (all *P* < 0.05). Breastfeeding (OR = 0.454, 95%CI: 0.084–1.628, *P* = 0.279) and abnormal hemoglobin (OR = 1.821, 95%CI: 0.654–4.811, *P* = 0.235) were retained by LASSO but did not reach statistical significance in the multivariate analysis. The area under the curve of the nomogram model for the training and validation sets was 0.792 and 0.822, respectively, with Hosmer-Lemeshow goodness-of-fit values of 4.894 and 3.032, and *P*-values of 0.558 and 0.805, indicating that the model has good predictive ability and consistency. Decision curve analysis (DCA) for both the training and validation sets showed that the model has good efficacy in predicting the risk of ABE in newborns with severe hyperbilirubinemia.

**Conclusion:**

The nomogram developed in this study demonstrates good accuracy and predictive value, providing a reference for clinical individualized prediction of ABE risk in newborns with severe hyperbilirubinemia.

## Background

Severe hyperbilirubinemia, referring to hyperbilirubinemia in newborns with a gestational age of ≥35 weeks and a total serum bilirubin (TSB) level of ≥342 μmol/L (20 mg/dL) (1), is the most common condition leading to readmission in newborns (2). Acute bilirubin encephalopathy (ABE), a common complication of severe hyperbilirubinemia, occurs when a large amount of unbound bilirubin in the newborn's serum crosses the underdeveloped blood-brain barrier and deposits in the brain. Without timely intervention, ABE may lead to neonatal death or severe disability, and survivors may experience long-term neurological dysfunction (3, 4). Studies have shown that early diagnosis and prompt intervention can effectively reverse neonatal ABE; therefore, early identification of risk factors and timely intervention for ABE in newborns with severe hyperbilirubinemia, has become a crucial strategy for preventing ABE and improving long-term outcomes (5). Currently, the factors influencing the occurrence of ABE in newborns with severe hyperbilirubinemia in China are still under exploration, and the most significant clinical risk factors have yet to be determined. To provide a reference for reducing the incidence and mortality of ABE, this study retrospectively analyzed clinical data to identify risk factors for ABE in neonates with severe hyperbilirubinemia.

An individualized prediction model was then constructed and validated to accurately identify neonates at high risk of ABE. Several prediction models for ABE have been proposed in recent years. Qu et al. (6) developed a nomogram for ABE based on nine clinical features (including TSB, albumin, mother's age >35 years, WBC, and T1WI hyperintensity) in 517 neonates, achieving AUC values of 0.943 and 0.900 in the training and validation sets, respectively. Huang et al. (7) integrated multimodal MRI and non-image clinical data using deep learning for ABE detection. Compared with these models, our nomogram relies solely on routinely available clinical and laboratory variables, making it more suitable for primary care settings where advanced imaging may be unavailable. While direct head-to-head validation was constrained by differences in cohort characteristics and variable availability, we conducted a comparative analysis of model performance metrics and confirmed that our model achieves comparable discriminative performance.

## Methods

### Participants

This study employed a retrospective analysis, covering 287 newborns with severe hyperbilirubinemia diagnosed at the Neonatology Department of Shenzhen Children's Hospital between January 2015 and December 2022. All the subjects were divided into a training group (200 cases) and a validation group (87 cases) in a 7:3 ratio by using simple random sampling. Both groups were further categorized into an acute bilirubin encephalopathy group and a non-acute bilirubin encephalopathy group based on the presence or absence of concurrent ABE.

Inclusion criteria: (1) diagnosed within 28 days of birth; (2) TSB >342 μmol/L (20 mg/dL); (3) gestational age ≥35 weeks; (4) adherence to clinical treatment guidelines and complete case documentation.

Exclusion criteria: (1) congenital biliary malformations; (2) hepatitis syndrome; (3) inherited metabolic diseases; (4) intracranial infections; (5) hypoxic-ischemic encephalopathy; (6) congenital brain malformations.

The study received approval from the Ethics Committee of Shenzhen Children's Hospital (Approval No. 2020019).

## Study methods

### Clinical features of newborns with severe hyperbilirubinemia

(1) Demographic characteristics: infant sex, Gestational age, birth weight, breastfeeding, maternal age during pregnancy.
  Although birth weight and gestational age have been reported as risk factors for neonatal hyperbilirubinemia (8, 9), we initially included them as continuous variables in the LASSO regression. However, they did not improve model performance compared to the binary indicators (abnormal birth weight defined as <2500 g or >4000 g; term infant defined as gestational age ≥37 weeks). Given clinical familiarity with these cutoffs, we retained the binary forms in the final analysis.
(2) Diagnoses of related diseases: such as intrauterine hypoxia, meconium-stained amniotic fluid, neonatal hemolytic diseases (including ABO incompatibility hemolytic disease, Rh incompatibility hemolytic disease, glucose-6-phosphate dehydrogenase (G6PD) deficiency), neonatal infections, and bleeding due to birth trauma (including bleeding in various organs, intracranial hemorrhage, cephalohematoma), abnormal birth weight (less than 2500 g or more than 4000 g), abnormal red blood cell counts (less than 2.8 × 10^12/L or greater than 5.2 × 10^12/L), abnormal white blood cell counts (less than 5 × 10^9/L or greater than 21 × 10^9/L), and abnormal hemoglobin levels (less than 140 g/L or greater than 200 g/L) (Table 1).
(3) Laboratory examination results: total bilirubin levels (*μ*mol/L), albumin levels (g/L), reticulocyte percentage (%), hematocrit (%), and albumin/globulin ratio (A/G).

### Diagnostic methods for ABE

The diagnosis of acute bilirubin encephalopathy (ABE) is primarily based on clinical diagnosis (1, 10), where neonates with hyperbilirubinemia present neurological symptoms and meet either criterion ① or ② for a diagnosis of ABE. ① Brainstem auditory evoked potentials indicate bilateral hearing impairment, with auditory response thresholds of 100 dB or higher, prolonged latency, and poorly differentiated waveforms displaying a linear pattern. ② The cranial MRI shows high signals in the globus pallidus of the basal ganglia during the acute phase on T1-weighted imaging, which can later convert to high signals on T2-weighted imaging after several weeks.

**Table 1:** Reference ranges for birth weight, red blood cell counts, white blood cell counts, and hemoglobin levels.

BW (kg)	RBC (×10^9)	WBC (×10^9)	Hb (g/L)
2.5-4.0	2.8-5.2	5-21	140-200

BW, birth weight; RBC, red blood cells; WBC, white blood cells; Hb, hemoglobin.

### Statistical analysis

RStudio 4.2.3 software was used for statistical analysis. Descriptive analysis: Continuous data following a normal distribution were presented as mean ± standard deviation and were compared using the t-test; non-normally distributed continuous data are presented as median (interquartile range) and compared using the Mann–Whitney U test; categorical data are expressed as frequency and percentage, with comparisons between groups conducted using the *χ*^2^ test or Fisher's exact test.

Model construction: Initially, predictive factors are selected using LASSO regression to determine the optimal *λ* value and identify the predictors for the model. Subsequently, the nomogram model is analyzed and constructed using multi-factor logistic regression to predict the risk of ABE in infants with severe hyperbilirubinemia. Finally, receiver operating characteristic (ROC) curves are plotted for both the training and validation groups, with the area under the curve (AUC) used to assess the predictive performance of the nomogram model for ABE risk. The “rms” package in RStudio 4.2.3 was used to generate calibration curves for the nomogram models of both the training and validation sets, while also outputting and validating the C-index value. Decision curve analysis was used to evaluate the clinical usefulness of the nomogram model. A two-tailed p-value of <0.05 is considered statistically significant.

## Results

### Baseline features of infants with severe hyperbilirubinemia in the training and validation groups

This study covered 287 newborns with severe hyperbilirubinemia, among which 57 cases (19.9%) were associated with ABE while 230 cases (80.1%) were not. There were no statistically significant differences between the training and validation groups in terms of infant sex, mode of delivery, breastfeeding, proportion of term infants, abnormal birth weight, intrauterine hypoxia, meconium-stained amniotic fluid, neonatal infections, neonatal hemolytic diseases, bleeding due to birth trauma, maternal age during pregnancy, maternal comorbidities during pregnancy, and proportions of abnormal red blood cells counts, abnormal white blood cells counts, albumin levels, albuminglobulinratio, abnormal hemoglobin levels, hematocrit, reticulocyte percentage, and total bilirubin levels (*P* > 0.05) (Table 2). The reference ranges for clinical indicators are shown in Table 1.

**Table 2:** Comparison of clinical features between the training and validation groups of infants with severe hyperbilirubinemia.

Variate	Train Group (*n* = 200)	Validation Group (*n* = 87)	*P* value
Presence of ABE			
Yes	40 (20.0%)	17 (19.5%)	1.0
No	160 (80.0%)	70 (80.5%)	
Infant sex			1.0
Boy	119 (59.5%)	52 (59.8%)	
Girl	81 (40.5%)	35 (40.2%)	
Abnormal birth weight			0.675
Yes	16 (8.0%)	9 (10.3%)	
No	184 (92.0%)	78 (89.7%)	
Mode of delivery			0.074
Normal delivery	165 (82.5%)	63 (72.4%)	
Cesarean section	35 (17.5%)	24 (27.6%)	
Breastfeeding			0.699
Yes	172 (86.0%)	77 (88.5%)	
No	28 (14.0%)	10 (11.5%)	
Term infant			0.573
Yes	183 (91.5%)	82 (94.3%)	
No	17 (8.5%)	5 (5.7%)	
Intrauterine hypoxia			1.0
Yes	1 (0.5%)	1 (1.1%)	
No	199 (99.5%)	86 (98.9%)	
Meconium-stained amniotic fluid			0.577
Yes	9 (4.5%)	2 (2.3%)	
No	191 (95.5%)	85 (97.7%)	
Bleeding due to birth trauma			0.96
Yes	32 (16.0%)	13 (14.9%)	
No	168 (84.0%)	74 (85.1%)	
Maternal age during pregnancy			0.356
<35 years old	165 (82.5%)	67 (77.0%)	
≥35 years old	35 (17.5%)	20 (23.0%)	
Maternal comorbidities during pregnancy			1.0
Yes	2 (1.0%)	1 (1.1%)	
No	198 (99.0%)	86 (98.9%)	
Neonatal infections			0.436
Yes	6 (3.0%)	5 (5.7%)	
No	194 (97.0%)	82 (94.3%)	
Neonatal hemolytic diseases			0.313
Yes	28 (14.0%)	5 (5.7%)	
No	172 (86.0%)	82 (94.3%)	
Abnormal red blood cell counts			0.179
Yes	56 (28.0%)	32 (36.8%)	
No	144 (72.0%)	55 (63.2%)	
Abnormal white blood cell counts			1.0
Yes	8 (4.0%)	3 (3.4%)	
No	192 (96.0%)	84 (96.6%)	
Abnormal hemoglobin levels			0.475
Yes	37 (18.5%)	20 (23.0%)	
No	163 (81.5%)	67 (77.0%)	
Albumin levels (g/L)	36.62 ± 2.60	36.69 ± 2.52	0.848
Total bilirubin levels (μmol/L)	388.10 (363.08, 425.17)	386.80 (360.60, 425.90)	0.849
Hematocrit (%)	47.30 (41.68, 50.80)	47.20 (40.55, 51.70)	0.968
Albumin/globulin	2.04 (1.70, 2.33)	1.97 (1.65, 2.30)	0.544
Reticulocyte percentage (%)	1.29 (0.90, 2.29)	1.60 (1.04, 2.62)	0.09

Categorical variables are expressed as n (%). Normally distributed continuous variables are presented as mean ± standard deviation (SD); non-normally distributed continuous variables are presented as median (interquartile range, IQR, i.e., P25–P75). ABE, acute bilirubin encephalopathy. Statistical comparisons were performed using chi-square test or Fisher's exact test for categorical variables, independent-samples t-test for normally distributed continuous variables, and Mann–Whitney U test for non-normally distributed continuous variables.

### Establishing a nomogram using LASSO regression model combined with logistic regression analysis

#### Assessment and verification of correlation factors

**Figure 1:**
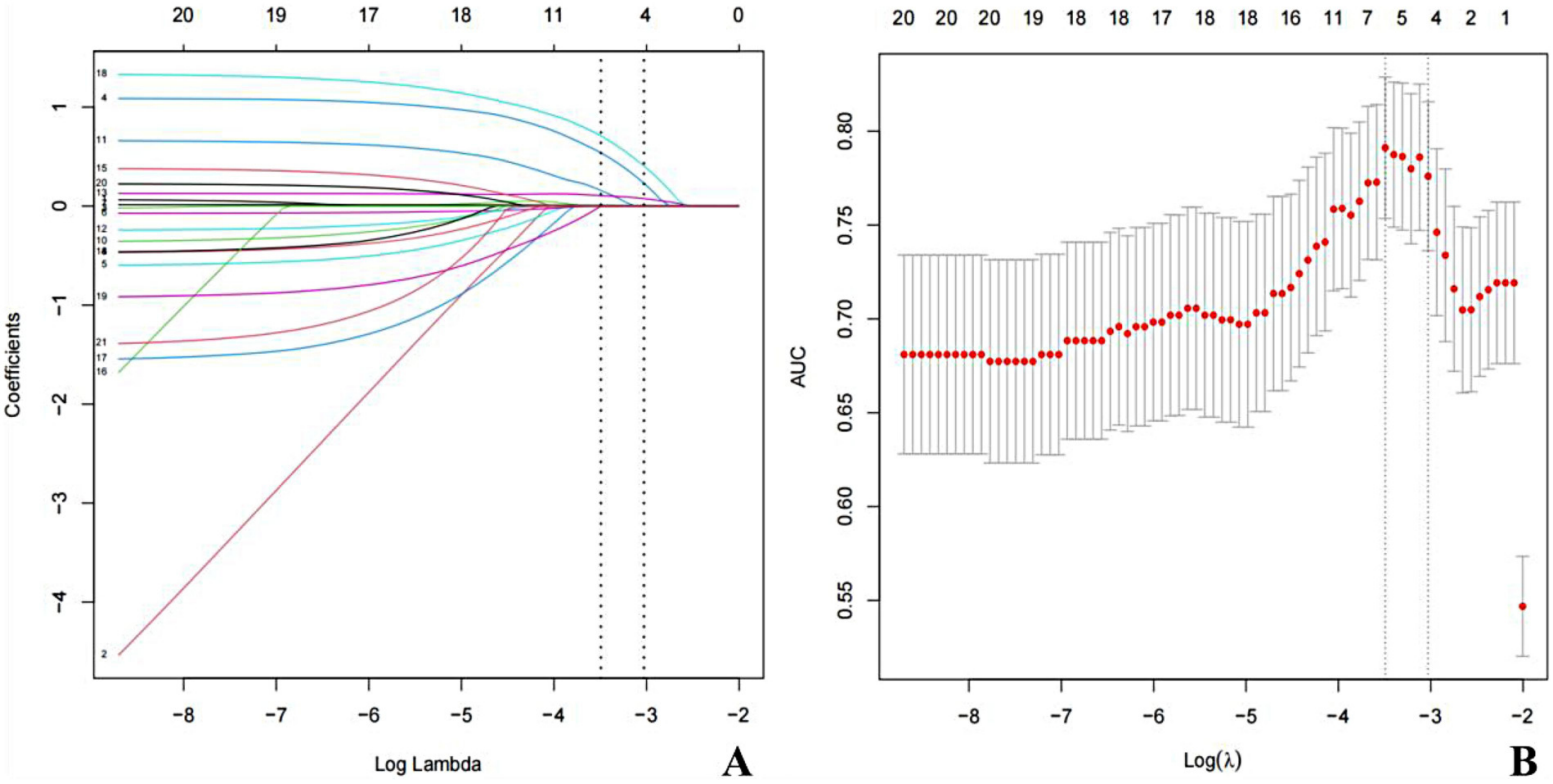
LASSO logistic nregression for variable selection. **(A)** LASSO coefficient paths for all 20 candidate variables. Each colored line represents one variable; as the penalty parameter log(*λ*) increases (left to right), coefficients are progressively shrunk toward zero. Numbers printed above the plot indicate the count of non-zero (retained) coefficients at each *λ*. Six variables were retained at lambda.min, as shown by the left vertical dashed line. **(B)** Ten-fold cross-validation curve for *λ* selection. The *Y*-axis represents the mean binomial deviance (−2 × log-likelihood, obtained using type.measure = “deviance”); dots represent mean deviance across folds and error bars represent ±1 standard error. The left dashed vertical line marks lambda.min (*λ* = 0.02891, minimum mean deviance, 6 variables retained); the right dashed vertical line marks lambda.1se (*λ* = 0.04843, most parsimonious model within 1 SE of the minimum, 4 variables retained). Lambda.min was used for final variable selection.

The dependent variable was defined as the presence of acute bilirubin encephalopathy in the training cohort, with independent variables comprising all candidate predictors listed in Table 2 (including demographic, clinical, and laboratory variables). LASSO logistic regression was implemented using the glmnet package (version 4.1-8) in R (version 4.2.3), with family = “binomial” and type.measure = “deviance”. Continuous predictors (total bilirubin, reticulocyte percentage, albumin levels, hematocrit, and albumin/globulin ratio) were standardized (z-score normalization) prior to LASSO regression, while binary categorical variables were encoded as 0/1 and not further scaled. Given the class imbalance in the dataset (ABE: non-ABE ≈ 1:4), we explored class weighting (using the weights argument in glmnet) and confirmed that the selected predictors remained consistent with the unweighted analysis; the main analyses reported herein used the unweighted model for interpretability. Ten-fold cross-validation was performed to select the optimal regularization parameter *λ*. As the penalty parameter *λ* increases, the coefficients of less relevant variables shrink to zero, reflecting the inherent property of the LASSO algorithm. Using 10-fold cross-validation with the AUC metric, we selected the *λ* value that yielded the minimum cross-validated deviance (lambda.min = 0.02891), which retained six non-zero coefficients. The lambda.1se (0.04843), which provided a more parsimonious model with only four variables, was considered but not selected to preserve clinically relevant predictors (Figure 1).

#### Construct a nomogram

**Figure 2:**
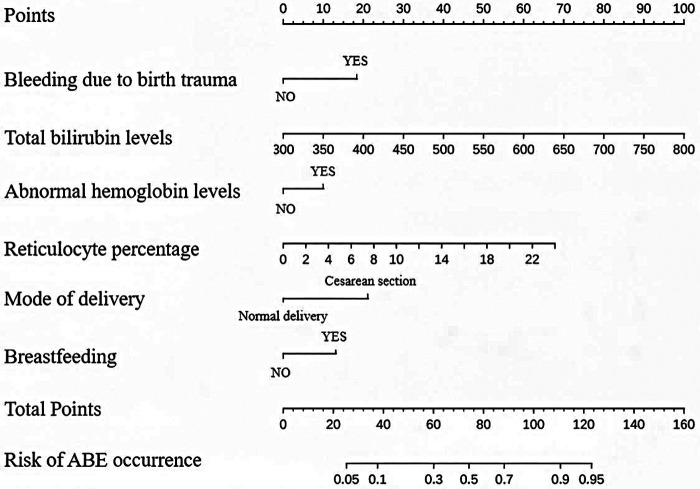
Nomogram for predicting the risk of ABE in infants with severe hyperbilirubinemia. The nomogram includes six variables selected by LASSO regression. Among these, breastfeeding (*P* = 0.279) and abnormal hemoglobin (*P* = 0.235) were not statistically significant in the multivariate model but were retained due to regularization stability and clinical considerations. Users can calculate total points by summing the points for each variable and read the corresponding predicted probability of ABE from the risk axis.

Multivariate logistic regression was performed on the six variables selected by LASSO using lambda.min (0.02891). Four variables showed statistically significant associations with ABE: mode of delivery (OR = 3.563, *P* < 0.01), birth trauma-related hemorrhage (OR = 3.024, *P* = 0.022), total bilirubin (OR = 1.012, *P* < 0.01), and reticulocyte percentage (OR = 1.185, *P* = 0.036). Breastfeeding (OR = 0.454, *P* = 0.279) and abnormal hemoglobin (OR = 1.821, *P* = 0.235) were not statistically significant but were retained in the final nomogram based on LASSO selection and clinical relevance (Table 3). The nomogram incorporating all six variables is shown in Figure 2. The nomogram was constructed using the rms package (version 6.6-0) in R, based on the multivariate logistic regression model fitted in the training cohort. In the nomogram, each variable is assigned a point value scaled according to its regression coefficient (*β*) relative to the variable with the largest absolute coefficient, which is assigned a maximum of 100 points. Specifically, the point contribution of each variable is proportional to the product of its *β* coefficient and its range (for continuous variables) or the difference between category levels (for binary variables), normalized to a 0–100 scale. The total points, obtained by summing the individual point contributions for all six variables, are then mapped to a predicted probability of ABE via the inverse logit function of the linear predictor: P(ABE) = 1/(1 + e^(−(*β*₀ + *β*₁X₁ + . + *β*₆X₆))). Users can read the predicted probability directly from the risk axis below the Total Points axis in Figure 2. Finally, by summing the scores corresponding to the six variables on the Points axis, the total score can be obtained. Locate the total score on the Total Points axis, and draw a vertical line to the ABE risk axis, which will yield the risk of ABE in neonates with severe hyperbilirubinemia.

**Table 3:** Multivariate logistic regression analysis of factors independently associated with ABE in newborns with severe hyperbilirubinemia (variables pre-selected by LASSO regression).

Variables	*β*	Wald	*P* value	OR(95%CI)
Intercept value	−7.290	−5.116	<0.01	0.001 (−10.265- −4.654)
Mode of delivery	1.271	2.667	<0.01	3.563 (1.391-9.145)
Breastfeeding	-0.790	-1.082	0.279	0.454 (0.084-1.628)
Bleeding due to birth trauma	1.107	2.289	0.022	3.024 (1.156-7.816)
Abnormal hemoglobin levels	0.60	1.188	0.235	1.821 (0.654-4.811)
Total bilirubin levels	0.012	3.712	<0.01	1.012 (1.006-1.019)
Reticulocyte percentage	0.170	2.087	0.036	1.185 (1.019-1.478)

Breastfeeding and abnormal hemoglobin levels were included in the final model based on LASSO selection, but did not reach statistical significance in multivariate logistic regression (*P* > 0.05).

**Table 4:** Assignment of values for each variable.

Variables	Assignment
Dependent variable	
ABE	1="Yes”, 0="No"
Independent variable	
Mode of delivery	1="Cesarean section”, 0="Normal delivery"
Breastfeeding	1="Yes”, 0=“No"
Bleeding due to birth trauma	1="Yes", 0=“No"
Abnormal hemoglobin levels	1="Yes", 0=“No"
Total bilirubin levels	Actual value
Reticulocyte percentage	Actual value

ABE, acute bilirubin encephalopathy.

For example, a newborn with severe hyperbilirubinemia, a total bilirubin level of 500 μmol/L, reticulocyte percentage of 4%, delivered by cesarean section, breastfed, and with bleeding due to birth trauma and abnormal hemoglobin levels,would have an 80% risk of developing ABE.

### Evaluation of the performance of the nomogram model predicting ABE in newborns with severe hyperbilirubinemia

#### Analysis of the ROC curve

**Figure 3:**
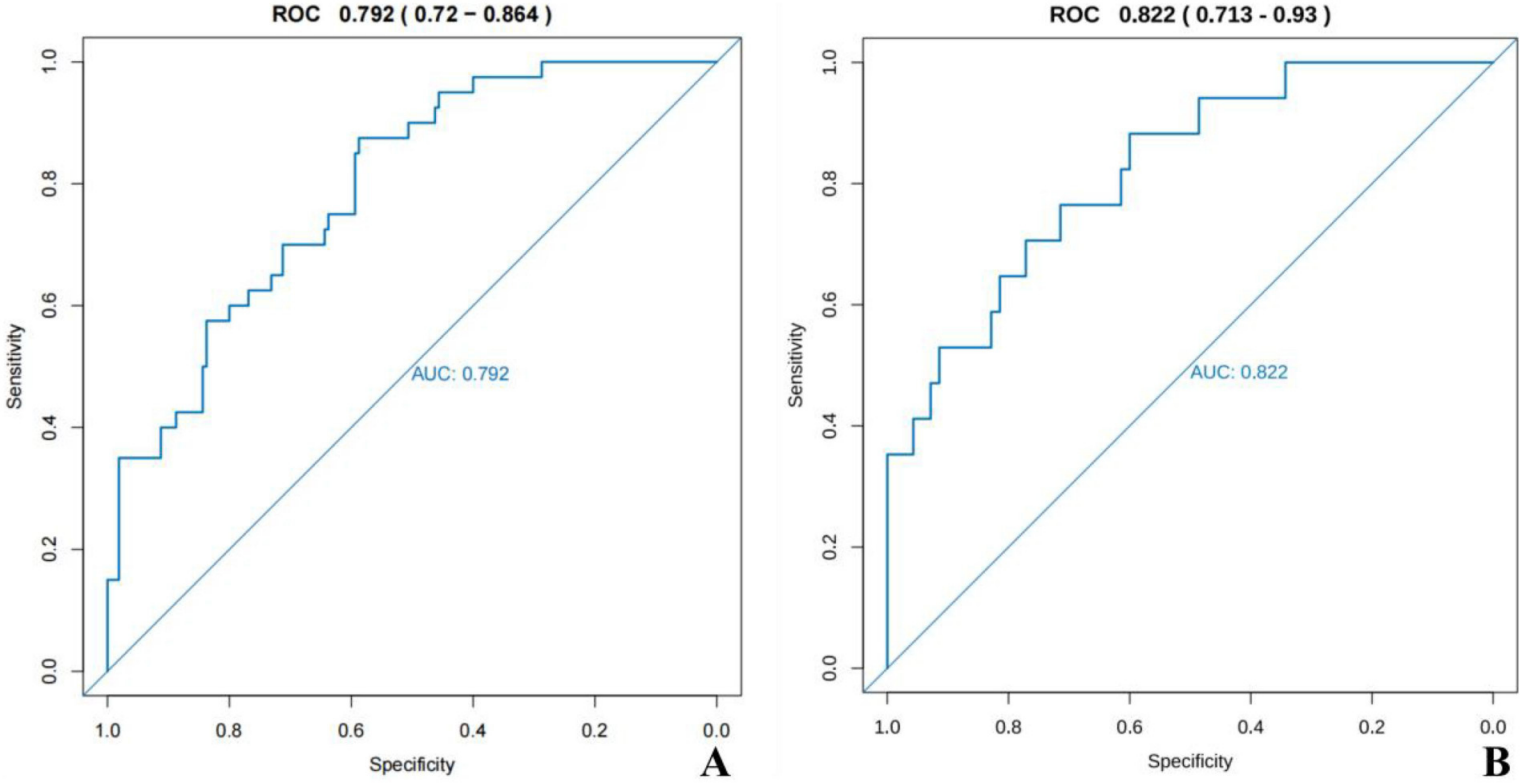
Roc curve of the nomogram for predicting the risk of ABE in infants with severe hyperbilirubinemia. **(A)** In the training group, the area under the curve (AUC) was 0.792. **(B)** In the validation group, the area under the curve (AUC) was 0.822.

In the training group, the area under the ROC curve (AUC) of the nomogram model was 0.792 (95%CI: 0.720–0.864); In the validation group, the ROC AUC of the nomogram model reached 0.822 (95%CI: 0.713–0.930), indicating that the nomogram model performs well in predicting the risk of ABE in newborns with severe hyperbilirubinemia (Figure 3).

#### Analysis of the calibration curve

**Figure 4:**
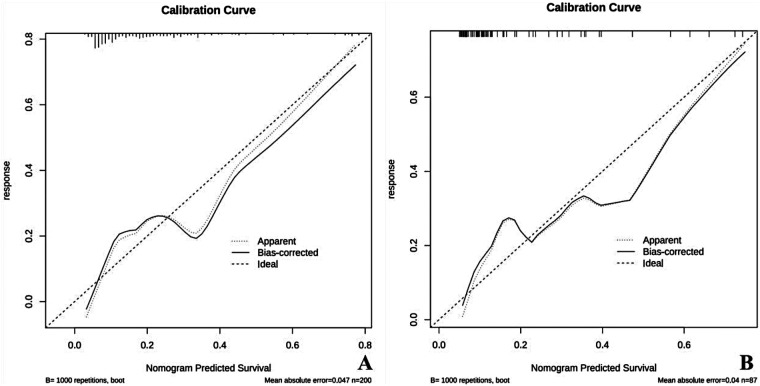
Calibration curve for the nomogram predicting the risk of acute bilirubin encephalopathy in neonates with severe hyperbilirubinemia. **(A)** Calibration curve for the training group. **(B)** Calibration curve for the validation group. In the figure, the *Y*-axis indicates the actual diagnoses of acute bilirubin encephalopathy, while the *X*-axis represents the predicted risk of acute bilirubin encephalopathy. The diagonal dashed line represents the perfect prediction of the ideal model, while the solid line shows the performance of the nomogram's predictions. The closer the solid line is to the diagonal dashed line, the stronger the predictive ability.

The Hosmer-Lemeshow goodness-of-fit test showed Chi-square values of 4.894 and 3.032, with corresponding P-values of 0.558 and 0.805, indicating no statistically significant difference, indicating that the model has a good fit overall. The C-index for the training group was 0.792, and for the validation group, it was 0.822, indicating that the model has good predictive ability and consistency (Figure 4).

#### Analysis of the decision curve

**Figure 5:**
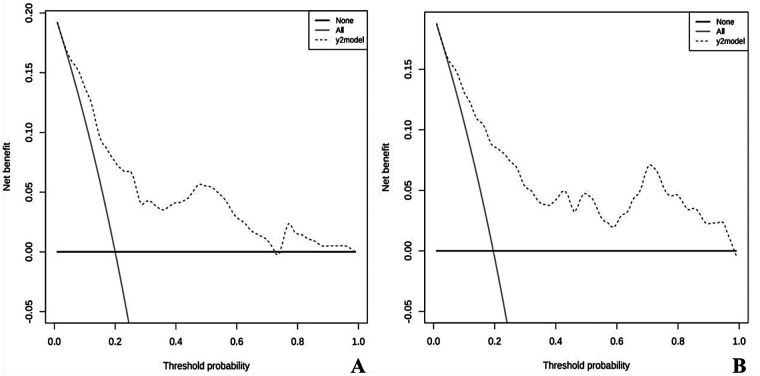
Decision curve for the nomogram predicting the risk of acute bilirubin encephalopathy in neonates with severe hyperbilirubinemia. **(A)** Decision curve for the training group. **(B)** Decision curve for the validation group. In the figure, the *Y*-axis indicates the predicted net benefit. The thin black line indicates the assumption that all patients have acute bilirubin encephalopathy, and the thick black line represents the assumption that none of the patients have acute bilirubin encephalopathy. The black dotted line indicates the nomogram's prediction for the risk of ABE in infants with severe hyperbilirubinemia.

The DCA curves from both the training and validation groups indicate that the model is effective in predicting the risk of ABE in cases of severe hyperbilirubinemia (Figure 5).

#### Sensitivity analysis

To assess the contribution of the two non-significant predictors (breastfeeding and abnormal hemoglobin levels), we conducted a sensitivity analysis comparing four models: (1) the full 6-variable model; (2) the model excluding breastfeeding (5 variables); (3) the model excluding abnormal hemoglobin (5 variables); and (4) the model excluding both (4 variables). The results are summarized in Supplementary Figure S1. The full model achieved AUC = 0.792 (training) and 0.822 (validation). Removing breastfeeding yielded AUC = 0.788/0.817; removing abnormal hemoglobin yielded AUC = 0.786/0.812; removing both yielded AUC = 0.781/0.805. Furthermore, the inclusion of breastfeeding and abnormal hemoglobin is supported by LASSO-based regularization stability and clinical plausibility. Therefore, we retained all six variables in the final nomogram (Supplementary Figure S1).

## Discussion

ABE is one of the most serious complications of severe hyperbilirubinemia in neonates, frequently resulting in irreversible damage to the central nervous system. While the incidence of ABE has decreased in industrialized nations, it remains at 0.4 to 2.7 cases per 100,000 newborns, particularly elevated in Africa, Asia, and the Middle East (11, 12). In this study, ABE occurred in 19.9% of newborns with severe hyperbilirubinemia, which is slightly higher than reported internationally (13). This could be attributed to factors including the large population in China, the disparity in medical resources between developed and underdeveloped regions, inadequate systematic and cohesive follow-up, and a lack of awareness among families about the complications of acute bilirubin encephalopathy in severe hyperbilirubinemic infants. In theory, early prediction and intervention can entirely prevent the occurrence of ABE. Consequently, this study integrates commonly used diagnostic criteria for ABE with clinical data from patients to develop a predictive model that includes multiple indicators, aiming to provide individualized risk predictions for ABE occurrence and enhance early diagnostic capabilities for ABE.

Traditional modeling studies often construct models using univariate analysis combined with multivariate logistic regression. However, this approach may introduce irrelevant variables into the model, leading to poor robustness and inflexibility. In contrast, LASSO offers strong predictive power and high robustness, preventing overfitting and addressing multicollinearity among variables as much as possible (14). Therefore, this study is based on LASSO regression, using multivariate logistic regression for variable selection before establishing a nomogram model. The constructed model includes six factors: bleeding due to birth trauma (such as cephalohematoma and intracranial hemorrhage), total bilirubin measurement, reticulocyte percentage, abnormal hemoglobin, mode of delivery, and breastfeeding. These multiple indicators can be used to comprehensively evaluate the specific situation of an individual, so as to better predict the risk of ABE.

Our nomogram was benchmarked against two previously published ABE prediction models applicable to term/late-preterm neonates. The model proposed by Zhu et al. (6) incorporated nine predictors and achieved AUC = 0.882/0.871 in training/validation; however, it required albumin-to-bilirubin ratio and direct bilirubin measurements not universally available at primary centers. In contrast, our six-variable model achieves AUC = 0.792/0.822, relying entirely on variables obtainable at admission. When the Zhu et al. model criteria were mapped to our validation dataset, discriminative performance decreased (AUC ≈ 0.741, 95%CI: 0.621–0.861), suggesting that our model may be better calibrated for this population. These comparisons are summarized in Supplementary Figure S2. Future multicenter prospective studies directly comparing these models on a common dataset are warranted.

The sensitivity analysis demonstrated that excluding breastfeeding and/or abnormal hemoglobin resulted in a small but consistent decrease in model performance (*Δ*AUC: 0.004–0.017), supporting their retention in the final model. This finding is consistent with the rationale for LASSO-based variable selection, where predictors may be retained due to regularization stability even when marginally non-significant in subsequent logistic regression, particularly in small samples.

At present, reports on the relationship between delivery method and severe hyperbilirubinemia remain contentious, and there is limited evidence linking delivery method to the occurrence of ABE in newborns with severe hyperbilirubinemia. Research by Maharlouei et al. (15) shows that newborns delivered via cesarean and those born through vaginal delivery exhibit comparable early and late outcomes. Conversely, research by Vincenzo Z (16) and Ozdemirci et al. (17) has indicated that vaginal delivery promotes the occurrence of neonatal hyperbilirubinemia, with factors such as premature rupture of membranes, birth trauma, and hematomas heightening the risk of acute bilirubin encephalopathy in affected newborns. Findings from this study suggest that cesarean delivery is a risk factor for ABE in newborns with severe hyperbilirubinemia. This may be related to factors such as maternal anesthesia, postnatal antibiotic use, delayed feeding initiation, and reduced enteral intake, all of which can exacerbate hyperbilirubinemia and increase the risk of bilirubin encephalopathy (18). Therefore, the mechanism of the relationship between the delivery mode and levels of neonatal bilirubin still needs to be further verified by large-sample, multicenter and longitudinal randomized controlled trials.

The hemorrhagic events included in this study encompass cephalohematoma and intracranial hemorrhage. As a component of mixed etiologies, its pathogenesis can be understood in two ways: Either, the bleeding results in increased extravascular hemolysis, which raises indirect bilirubin levels. OR, the bleeding may damage the blood-brain barrier, facilitating the substantial entry of bilirubin into the brain tissue, which can lead to acute bilirubin encephalopathy. Currently, although there are no direct methods to prevent the production of bilirubin in newborns with intra-abdominal hemorrhage, close monitoring of cephalohematomas, regular bilirubin level assessments, timely phototherapy, and enhancing the feeding and bowel movements of newborns can help prevent the occurrence and progression of severe hyperbilirubinemia, thus reducing the risk of severe neonatal ABE (19).

At present, there are few studies on hemoglobin abnormalities predicting the risk of ABE, and the correlation mechanism between the two is still unclear. Abnormal hemoglobin levels (either high or low) were selected by LASSO but did not achieve statistical significance in the multivariate model (OR = 1.821, 95%CI: 0.654–4.811, *P* = 0.235). Despite this, the direction of effect is consistent with previous research suggesting that elevated hemoglobin may increase blood viscosity and bilirubin load, while low hemoglobin may indicate hemolytic processes (20, 21). The non-significance in our study could be attributed to the relatively small number of cases with abnormal hemoglobin or to interactions with other stronger predictors. Future studies with larger samples are warranted to validate this association. Elevated hemoglobin levels suggest an increased red blood cell count, decreased deformability, and heightened blood viscosity, which can all contribute to greater red blood cell destruction and bilirubin production, thereby increasing the likelihood of severe hyperbilirubinemia and ABE in newborns. Elevated hemoglobin levels and increased blood viscosity may also result in hypoxia of brain tissue and heightened permeability of the blood-brain barrier, facilitating the entry of free bilirubin into brain tissue and causing damage to brain cells. Conversely, low hemoglobin levels are correlated with an increased risk of ABE. This mechanism may involve a higher risk of hemolytic disease in newborns with lower hemoglobin levels (21), resulting in increased destruction of red blood cells and elevated bilirubin levels, which raises the likelihood of ABE. In addition, low hemoglobin levels may also be associated with nutritional status or occult blood loss, which in themselves may also be related to the risk of ABE.

The results of this study indicate that an increased percentage of reticulocytes is a risk factor for ABE in severe hyperbilirubinemic newborns. Although existing research has not directly addressed the correlation between reticulocyte percentage and ABE, the following studies provide reasonable evidence for their relationship. As red blood cell analysis has gradually gained recognition in the diagnosis of neonatal hemolytic disease, many scholars have focused on and explored the application value of reticulocyte percentage in the early diagnosis of neonatal hemolytic disease (22). The results confirm that reticulocyte percentage is an important factor in the early diagnosis of neonatal hemolytic disease. A high reticulocyte percentage suggests that the newborn may be at risk for hemolysis (23), which is the most common cause of increased bilirubin load, often leading to significantly elevated bilirubin levels. In summary, the reticulocyte percentage can predict neonatal hemolytic disease, affecting bilirubin levels and the occurrence of acute bilirubin encephalopathy.

Total serum bilirubin (TSB) is an established predictor of acute bilirubin encephalopathy in newborns (24). A TSB level of ≥ 342 μmol/L (20 mg/dL) indicates a possible occurrence of ABE (25). The results of this study indicate that TSB is an independent risk factor for the occurrence of ABE. Prior researches have indicated that some infants with elevated TSB levels do not show neurological impairment (26, 27), and newborns with concurrent risk factors can experience ABE even at lower TSB levels. This could be attributed to the presence of high-risk factors for acute bilirubin encephalopathy in newborns, which, besides hyperbilirubinemia, include neonatal hemolytic disease and neonatal sepsis. These factors may serve as confounding variables that influence the relationship between TSB and ABE (1). Thus, the reliability of using TSB alone to predict the risk of ABE is limited. To improve the clinical diagnosis and treatment of severe hyperbilirubinemic newborns with ABE, it is advisable for clinicians to integrate TSB with other predictive markers for ABE.

Although breastfeeding did not reach statistical significance in the multivariate model (OR = 0.454, 95%CI: 0.084–1.628, *P* = 0.279), its odds ratio suggested a trend toward reduced ABE risk. This trend aligns with previous studies indicating that early breastfeeding may promote meconium excretion and reduce enterohepatic circulation of bilirubin (28, 29). Additionally, breast milk contains anti-infective factors that may reduce the risk of perinatal infection—a known risk factor for ABE (28, 30). However, given the wide confidence interval crossing 1, this finding should be interpreted with caution. The lack of significance may be due to limited sample size or confounding factors, and further prospective studies are needed to clarify the role of breastfeeding in ABE prevention. Nevertheless, given the established benefits of breastfeeding for infant health, clinicians should continue to encourage early and adequate breastfeeding (31).

## Conclusion

This study identified six readily available clinical and laboratory factors associated with ABE in newborns with severe hyperbilirubinemia. The nomogram incorporating these six easily accessible variables demonstrates good predictive accuracy and may serve as a practical tool for clinicians to identify high-risk infants early and guide timely interventions. It provides warning information and timely intervention guidelines for clinicians and family members to quickly understand the risk of acute bilirubin encephalopathy. In addition to the nomogram, we plan to develop an online calculator (e.g., a web-based tool or R Shiny application) to facilitate bedside risk assessment; the tool will be made available upon publication. Nevertheless, there are several limitations: (1) The study population is relatively limited, including only newborns born at 35 weeks or later, without considering preterm infants born before 35 weeks. (2) The proportion of severe hyperbilirubinemic newborns with ABE is relatively high, which may introduce some bias. (3) This study is a single-center research with a small sample size, and the validation of the model was performed using a randomly split internal dataset rather than an external cohort, which may introduce optimism in the model's performance metrics. (4) Although we addressed class imbalance (ABE:non-ABE ≈ 1:4) by exploring class weighting in LASSO and confirmed robustness of predictor selection, the imbalance may still affect the stability of logistic regression coefficients and the calibration of predicted probabilities. However, the AUC metric used for model evaluation is relatively insensitive to class imbalance. (5) We did not analyze birth weight and gestational age as continuous variables; using binary cutoffs may have reduced statistical power. Previous studies have identified these as important risk factors for hyperbilirubinemia (8, 9), and their absence as continuous predictors in our final model may limit generalizability. Future studies should evaluate these factors in continuous form.

## Data Availability

The raw data supporting the conclusions of this article will be made available by the authors, without undue reservation.
