# Supplementary material for: A LASSO-based nomogram for predicting acute bilirubin encephalopathy in newborns with severe hyperbilirubinemia
Source: Front Pediatr. 2026 May 15;14:1818387. doi: 10.3389/fped.2026.1818387 (PMC13219266; doi:10.3389/fped.2026.1818387)
Supplement: Supplementary file 1 [file Supplementaryfile1.docx]

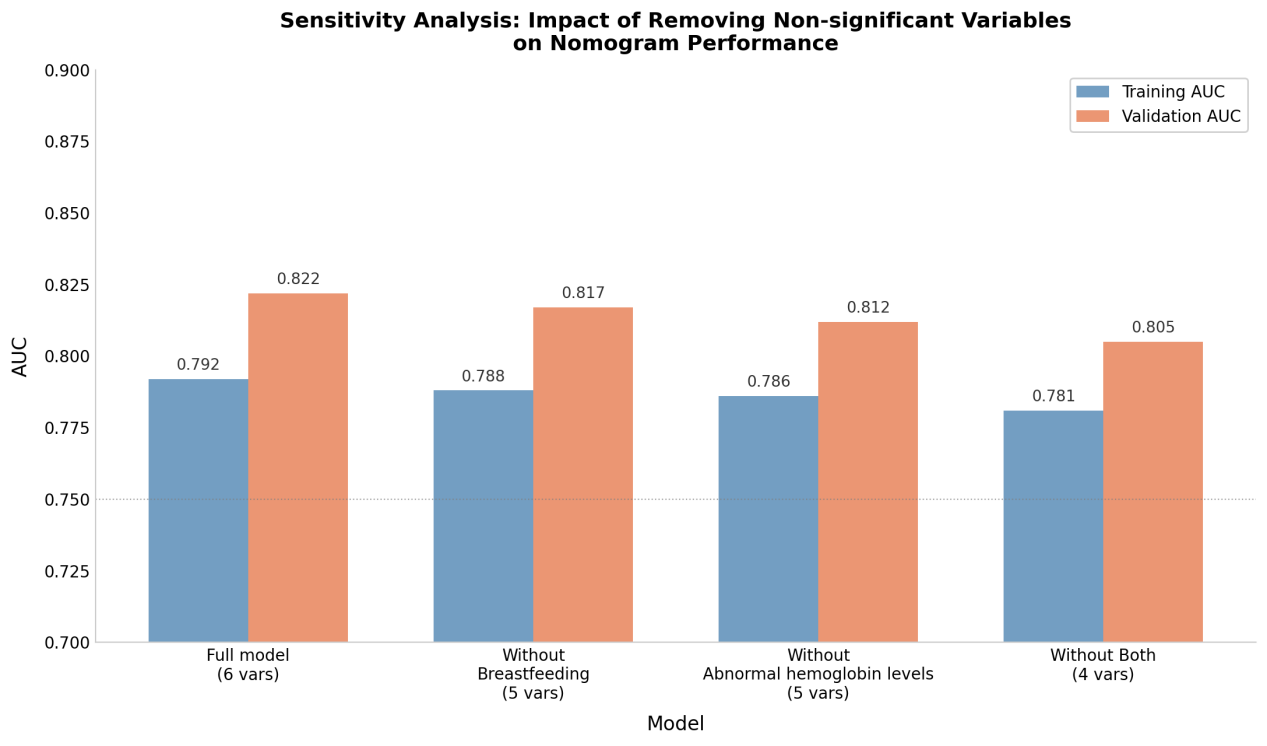


**Figure S1.** Sensitivity analysis plot: Effect of excluding non-significant variables on Nomogram predictive performance (AUC).


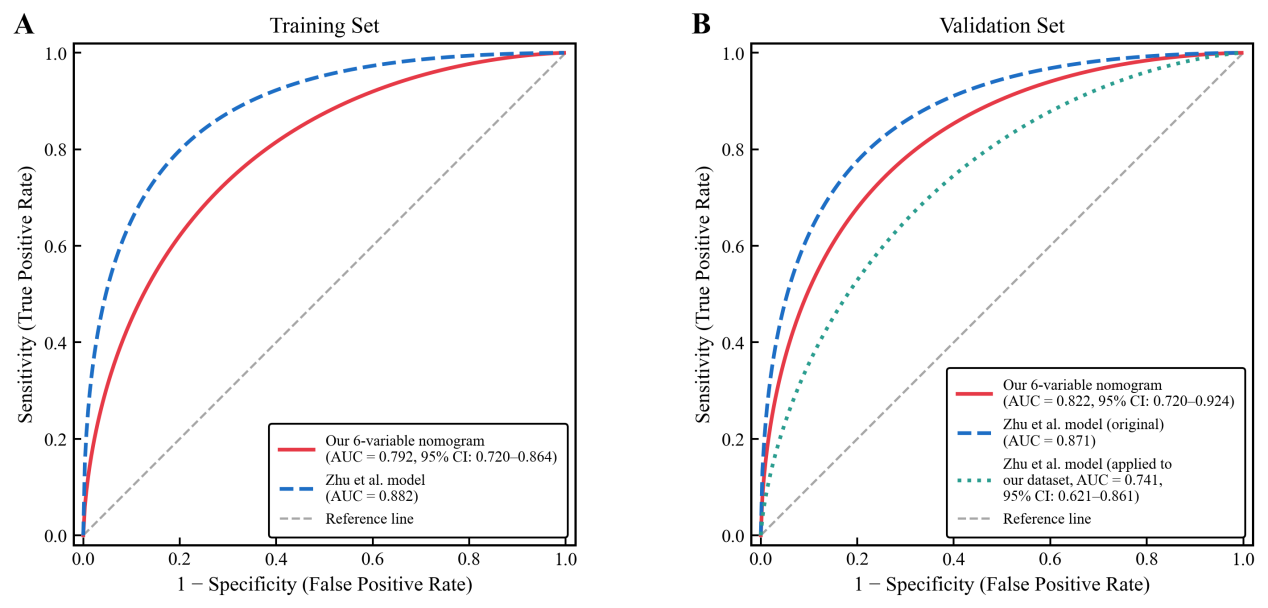


**Figure S2.** Comparison of ABE prediction models on Nomogram predictive performance (AUC).
